# Initial detections and spread of invasive *Spodoptera frugiperda* in China and comparisons with other noctuid larvae in cornfields using molecular techniques

**DOI:** 10.1111/1744-7917.12700

**Published:** 2019-07-21

**Authors:** Da‐Peng Jing, Jing‐Fei Guo, Yu‐Ying Jiang, Jian‐Zhou Zhao, Amit Sethi, Kang‐Lai He, Zhen‐Ying Wang

**Affiliations:** ^1^ State Key Laboratory for Biology of Plant Diseases and Insect Pests Institute of Plant Protection, Chinese Academy of Agricultural Sciences Beijing China; ^2^ China National AgroTech Extension and Service Center Beijing China; ^3^ Corteva Agriscience Johnston IA USA

**Keywords:** corn, molecular identification, *Mythimna separata*, *Spodoptera exigua*, *Spodoptera frugiperda*, *Spodoptera litura*

## Abstract

The fall armyworm, *Spodoptera frugiperda*, is a species native to the Americas and has spread to many countries in Africa and Asia in recent years. Proactive actions for potential invasion of *S. frugiperda* to China coordinated by government agencies and agricultural extension systems resulted in timely detection in January 2019 in Yunnan province neighboring onto Myanmar. The extensive monitoring in southern provinces of China since February 2019 resulted in dynamic tracking of *S. frugiperda* spreading to 13 provincial regions in China within 4 months by May 10, 2019, which is crucial for timely management actions in the fields. The first detections of *S. frugiperda* (corn strain) in China were confirmed using cytochrome oxidase subunit 1 (CO1) and triosephosphate isomerase (*Tpi*) genes molecular marker method. In addition to *S. frugiperda*, larvae of three other noctuid species with similar morphological appearance (*S. litura*, *S. exigua* and *Mythimna separata*) can occur simultaneously and cause similar damage in cornfields in southern China. Thus, we can use both morphological and molecular marker methods to compare larval stages of four noctuid species. Further, we discuss the risk of potential spread of invasive *S. frugiperda* to other regions and impact on corn production in China.

## Introduction

Fall armyworm, *Spodoptera frugiperda* (J.E. Smith) (Lepidoptera: Noctuidae), is one of the most devastating pests of corn in the Americas. *S. frugiperda* is a polyphagous pest with a broad range of hosts, over 80 plant species, and it is highly migratory (Pogue, 2002; CABI, 2016). It is known that *S. frugiperda* feeds on corn leaves, tassel and ears during the larval stage, which causes serious damage to corn (Sena *et al*., 2003). In Brazil, *S. frugiperda* attacks on corn reduced the yield up to 34%, causing annual losses of 400 million US dollars (Sena *et al*., 2003; Figueiredo *et al*., 2005; Lima *et al*., 2010). It is native to tropical and sub‐tropical areas in America but has spread rapidly across almost all sub‐Saharan countries after it was first detected in western and central Africa in early 2016, and 2018 in Uganda (Goergen *et al*., 2016; Otim *et al*., 2018). Then it was confirmed in several Asian countries such as India (Mallapur *et al*., 2018), Sri Lanka, Thailand, Yemen and Myanmar (CABI, 2019) as well as Bangladesh (Farmer, 2019) in 2018.

When *S. frugiperda* invasion was confirmed in India in July 2018, we submitted a report “Rapid spread of crop‐devastating fall armyworm in Africa and invasion in India” to the government agencies of Ministry of Agriculture and Rural Affairs (MARA), China in August 2018, and then published a review paper “Potential invasion of the crop‐devastating insect pest fall armyworm *Spodoptera frugiperda* in China” in *Plant Protection* in November 2018 (Guo *et al*., 2018). When *S. frugiperda* was confirmed in Myanmar in December 2018, the notification was issued by MARA for field monitoring in Yunnan and Guangxi provinces neighboring Myanmar for timely detection of *S. frugiperda*. The first suspected detection of *S. frugiperda* in China was confirmed based on morphological methods in January 2019 [(China National Agro‐Tech Extension and Service Center (NATESC), 2019a; International Plant Protection Convention (IPPC, 2019)] in Yunnan province adjacent to Myanmar. It was also confirmed by methods using molecular makers (Zhang *et al*., 2019).

The females lay eggs on abaxial leaf surfaces of host leaves and an individual adult can lay 100 to 200 eggs per egg mass (Kumela *et al*., 2018). Immediately after hatching, neonates start feeding on the leaf which leads to complete skeletonization (Brévault *et al*., 2018). Duration of larval stage (usually six instars) is about 14 days during the summer and may prolong to 30 days during cold weather and the last instar moves onto the ground and pupates (Sparks, 1979). The adult of *S. frugiperda* has strong flight ability and has spread rapidly in Africa, India and other Asian countries (Early *et al*., 2018).

To control *S. frugiperda*, an effective identification method is needed because similar species like *S. litura* (Fabricius), *S. exigua* (Hübner) and *Mythimna separata* (Walker) also occur simultaneously in cornfields. In our previous report, the morphological differences among the four noctuid species were described (Guo *et al*., 2019). However, the morphological characteristics of these pests and the damage symptoms they cause on corn leaves are very similar, and sometimes this may lead to misidentification with other noctuid species, especially for the 1st and 2nd instars. Also, egg masses of the three *Spodoptera* species are very difficult to distinguish based on morphological characteristics. In addition, *S. frugiperda* consists of “rice” and “corn” subpopulations and differs in host plant distribution without difference in morphological features (Pashley, 1986; Pashley *et al*., 1987; Nagoshi *et al*., 2017). Molecular identification methods could be more accurate than the morphological identification. With the rapid advancement in molecular biology techniques, various biological macromolecules containing evolutionary information have been used as molecular markers, which are widely used in species identification and phylogenetic analysis (Tautz *et al*., 2002). Mitochondrial DNA (mtDNA) has many advantages for intraspecific identifications due to maternal inheritance, high copy number, and both conservative and high‐mutation rate DNA segments (Mauro *et al*., 2006). Currently, the cytochrome oxidase subunit 1 (*CO1*) gene has been used as a standard universal sequence used to elucidate the species uncertainty in morphological taxonomy (Paul *et al*., 2003; Murray & Prowell, 2005; Solovyeva *et al*., 2014) and this technology has been adapted to distinguish *S. frugiperda* (Nagoshi, 2007a; Nagoshi, 2007b; Kergoat *et al*., 2012; Cock *et al*., 2017). In addition, polymorphisms in triosephosphate isomerase (*Tpi*) gene (Knowles, 1991) helps to identify specific host strains and the efficiency of this gene during species identification has been explained in many reports (Nagoshi, 2010, 2012; Nagoshi *et al*., 2017).

In this study, we reported the initial detections and sequential spread of *S. frugiperda* in 13 provincial regions (Yunnan, Guangxi, Guangdong, Guizhou, Hunan, Hainan, Fujian, Zhejiang, Hubei, Sichuan, Jiangxi, Chongqing, and Henan) of China within 4 months by May 10, 2019 and confirmation of the species using molecular markers based on *CO1* and *Tpi* genes. We also compared molecular differences among four similar noctuid larvae of *S. frugiperda*, *S. exigua*, *S. litura* and *M. separata* from cornfields.

## Materials and methods

### Initial detections of S. frugiperda in China

On January 3, 2019, NATESC (2019b) of MARA published a notification to provincial Plant Protection Stations nationwide in China for proactive actions for potential invasion of *S. frugiperda* into China, with special requirement to set up key monitoring sites for all year‐round monitoring in the four southern provinces (i.e., Yunnan, Guangxi, Guangdong, Hainan) and other regions with high invasion risks. The major monitoring methods were: (1) on‐farm monitoring by national agricultural extension agencies, National Crop Pest Monitoring and Forecasting Regional Stations (>1000 in total), with special attention in winter cornfields in Yunnan and Guangxi starting January 2019, in the southern provinces starting February 2019, and nationwide starting April 2019; (2) adult trapping using black light traps and vertical‐pointing searchlight traps in the southern provinces starting February 2019 and 220 counties of 26 provinces since April 2019; (3) sex pheromone trapping using equipment confirmed by NATESC on key host crops in the four southern provinces since February 2019. NATESC published a standardized method “Methods of Investigation and Forecast for Fall Armyworm in 2019 (Preliminary)” in March 2019 for data collection in China (NATESC, 2019c).

### Sample collection and molecular identification and analysis of CO1 and Tpi genes

In January, 2019 the first suspected *S. frugiperda* infestation was detected in a cornfield in Jiangcheng county (of Pu'er City), Yunnan province; we surveyed *S. frugiperda* on winter corn in Pu'er and Dehong (Dehong Dai and Jingpo Autonomous prefecture) of Yunnan province during January 12–24 and confirmed the *S. frugiperda* invasion by morphological identification. The *S. litura*, *M. separata* and *S. exigua* larvae, as well as suspected *S. frugiperda* larvae were collected from a winter cornfield in Dehong, Yunnan province (24.42°N, 98.60°E) on January 20, 2019. Totally, 250–350 larvae were collected from Yunnan province. The collected larvae were transported to the laboratory and reared in a controlled environment chamber at 27±1 °C, with 60%–70% relative humidity (RH), a 14​:​10 L​:​D photoperiod.

**Figure 1:**
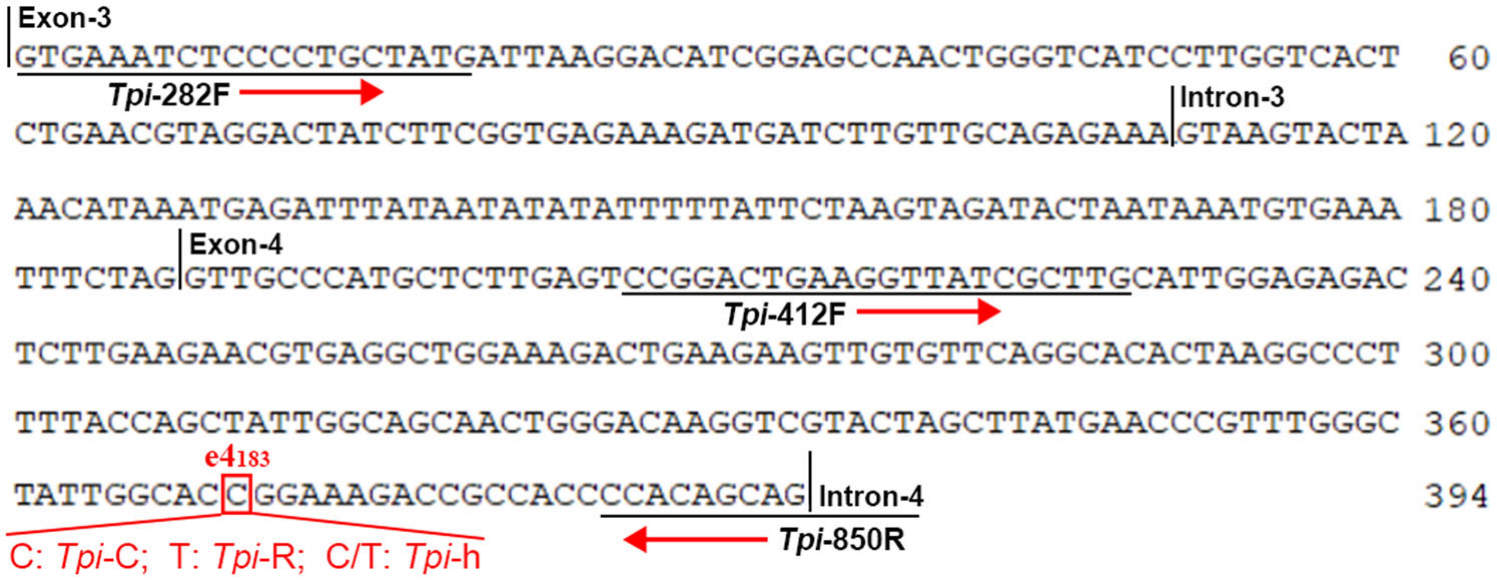
Triosephosphate isomerase (*Tpi*) gene mapping at e4_183_ polymorphic site for a C or T nucleotide. Polymerase chain reaction amplification was done using primers *Tpi*‐282F and *Tpi*‐850R. DNA sequencing was performed using primer *Tpi*‐412F, which initiates in the same exon as e4_183_. [Correction added on 12 September 2019, after first online publication: Figure 1 has been corrected to include a missing ‘C’ in the sequence.]

The CO1 primer of *S. frugiperda*, *M. separata* (Mehrdad *et al*., 2006) and *S. exigua* (Hebert *et al*., 2003) were designed based on previous reports. Total DNA was extracted from whole body of 4th instars using a DNA kit (Tianmoboi, China) and stored at −20 °C for further analysis. Twenty individuals from each species were selected and genomic DNA was extracted from every individual larva (*n* = 20). Polymerase chain reaction (PCR) amplifications were carried out in a volume of 20 *µ*L reaction containing: 10 *µ*L of Super HF PCR Master Mix (HeroGen Biotechnology, USA), 2 *µ*L primers (Forward and Reverse), 1 *µ*L DNA template and 7 *µ*L sterile H_2_O. *S. litura* primers were designed according to the sequence from the National Center for Biotechnology Information (NCBI) (GenBank No. KF022223.1). On other hand, based on single‐nucleotide polymorphisms at e_4_183 site the *Tpi* gene was selected for analysis and this site explains strain differentiation. In females, the *Tpi* e_4_183 site possesses either a C or T nucleotide, which can differentiate the two *S. frugiperda* strains. The corn strain possesses C nucleotide (*Tpi* e_4_183‐C) and rice strain with T (*Tpi* e_4_183‐R). These *Tpi* genes are likely to be Z‐linked in Lepidoptera (Yasukochi *et al*., 2006). However, there are more possibilities of overlap between *Tpi* genes in male genomic DNA during PCR product sequencing, which possess both C and T nucleotides at e_4_183 site (Nagoshi, 2010; Nagoshi *et al*., 2017). To overcome this problem, primer design and methods were followed according to Nagoshi *et al*. (Fig. 1) (Nagoshi, 2010; Nagoshi *et al*., 2017). Twenty larvae were selected randomly, and DNA was isolated. All specific primer details are mentioned in Table 1. Amplifications were performed in Techne‐5000 (UK) and programmed according to the following conditions: initial denaturation at 94 °C for 5 min; 30 amplification cycles consisting of 1 min at 94 °C, 30 s at 50 °C, 45 s of extension at 72 °C, and final extension at 72 °C for 10 min.

**Table 1:** Selected species‐specific cytochrome oxidase subunit 1 and triosephosphate isomerase (*Tpi*) primers

Species	Primer	Sequence (5′–3′)
*Spodoptera frugiperda/Mythimna separata*	Forward	ATTCAACCAATCATAAAGATATTGG
	Reverse	TAAACTTCTGGATGTCCAAAAAATCA
*S. exigua*	Forward	GGTCAACAAATCATAAAGATATTGG
	Reverse	TAAACTTCAGGGTGACCAAAAAATCA
*S. litura*	Forward	ACAAATCATAAAGATATTGGAACATTA
	Reverse	TAAACTTCGGGATGTCCAAAAAATCA
*Tpi*‐282F	–	GGTGAAATCTCCCCTGCTATG
*Tpi*‐850R	–	AATTTTATTACCTGCTGTGG
*Tpi*‐412F	–	CCGGACTGAAGGTTATCGCTTG

### Sequencing and species identification

The PCR products from four noctuid species were outsourced to Shenggong Biotec (Shanghai, China) for sequencing. The assembled sequences from four samples were subjected to molecular identification using the online NCBI Basic Local Alignment Search tool (BLAST) (https://www.ncbi.nlm.nih.gov/). The assembled nucleotide using the CO1 method was submitted to NCBI (GenBank No. MK790611). A phylogenetic tree was constructed using the maximum likelihood method (MEGA 7.0 software, USA) with 1000 replications.

## Results

### The initial detection of S. frugiperda in China

#### Larvae detections

**Figure 2:**
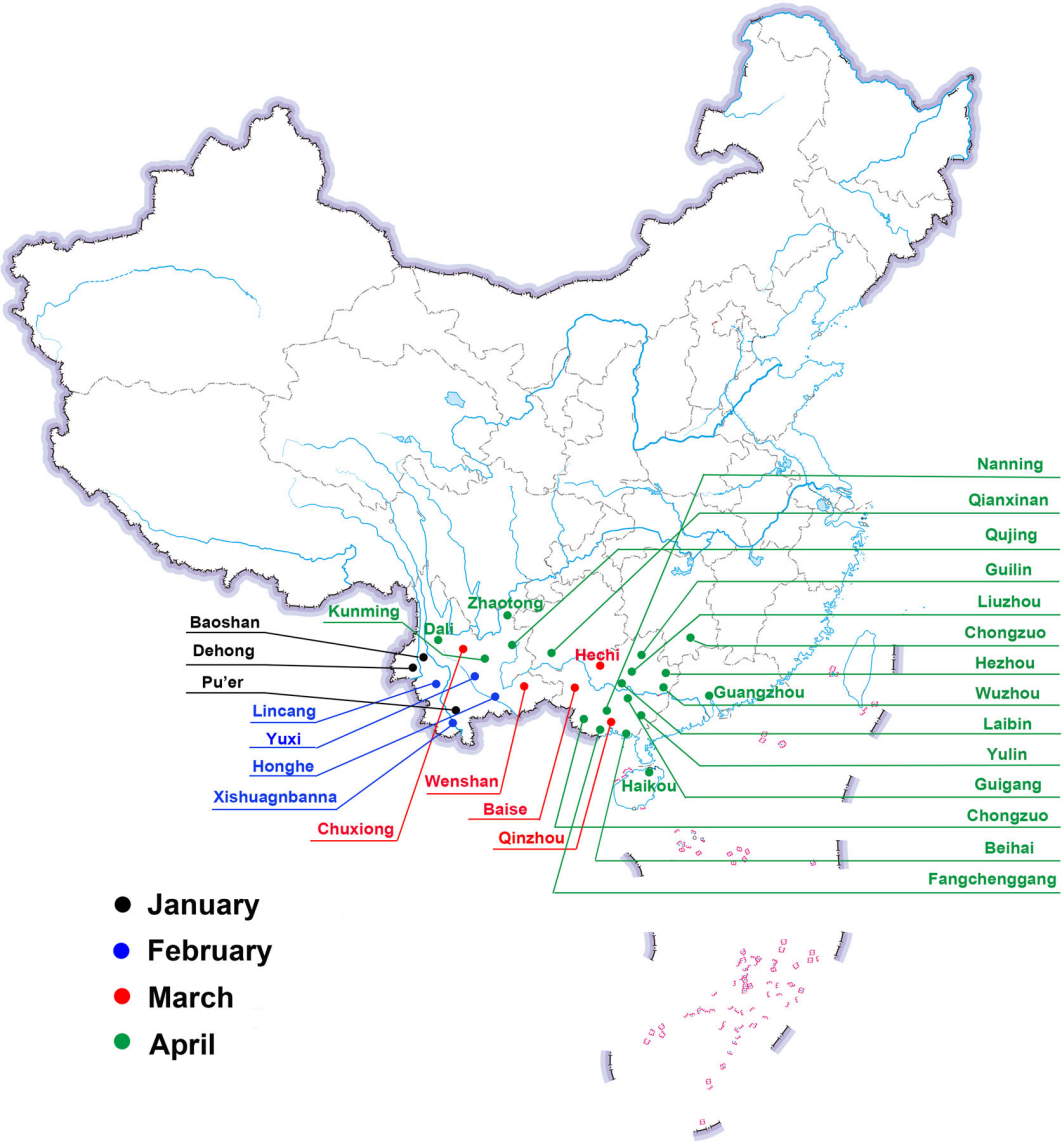
Map showing the location of *Spodoptera frugiperda* in China by April 30, 2019. The dots in black, blue, red and green represent areas where *S. frugiperda* were found in respective months of 2019. GS(2019)3426.

The first suspected *S. frugiperda* infestation was detected in a cornfield in Jiangcheng county, Yunnan province on January 11, 2019. Additional field surveys on wide range areas in Yunnan province confirmed the presence of *S. frugiperda* larvae in cornfields in three cities or autonomous prefectures (Pu'er, Dehong and Baoshan) of Yunnan province by the end of January 2019. There was an average of 0.05–0.20 larvae per corn plant and maximum two larvae per plant. There were six cities or autonomous prefectures in Yunnan with *S*. *frugiperda* larvae in cornfields by the end of February 2019 (Fig. 2). By April 2019, there were 63 counties from 12 cities or autonomous prefectures in Yunnan with the infestation of larvae detected on 5200 ha of corn fields and 3700 ha in sugarcane fields, with highest larvae infestation of 100% corn plants and up to 1.5–2.0 larvae per plant in selected fields in Longchuan county.

By the end of April 2019, there were six provincial regions in China with *S*. *frugiperda* larvae detected (Fig. 2); Guangxi Zhuang Autonomous Region was the second provincial area with *S. frugiperda* larvae detected in two cities (Chongzuo and Baise) by April 2019. The highest larvae infestation was 50% of corn plants in selected cornfields. Guangdong was the third province with *S. frugiperda* larvae detected in Zengcheng and Conghua districts of Guangzhou City. In late April 2019 the *S. frugiperda* larvae were detected in the fourth and fifth provincial regions of Guizhou (Qianxinan Prefecture) and Hunan province (Chenzhou City). On April 30, 2019 the *S. frugiperda* larvae were detected in Haikou of Hainan as the sixth province, which is the major region of winter research nurseries for corn breeding in China. By May 10, 2019, it was also found in seven additional provincial regions (Fujian, Zhejiang, Hubei, Sichuan, Jiangxi, Chongqing and Henan) resulting in 13 provincial regions in total within four months (Table 2).

**Table 2:** The severity of *Spodoptera frugiperda* infestations in 13 provinces in China by May 10, 2019 (NATESC, 2019e, f)

Provincial region	Month of first detection	Insect stage	No. of cities	No. of counties	Crop type	Infestation and crop acreage
Yunnan	January	Larvae	15	112	Corn	20 000 ha, up to 3 larvae per plant and normally 1%–10% plants infested.
	April	Larvae	3	3	Sugarcane	3680 ha, up to 40% plants infested in Longchuan county.
Guangxi	March	Adults	3	6	–	–
	April	Larvae	14	65	Corn/sugarcane	Corn: 46 600 ha, up to 6 larvae per plant and normally 5%–9% plants infested.
Guangdong	April	Larvae	7	16	Corn	187 ha, up to 0.2 larvae per plant and 10%–20% plants infested.
Guizhou	April	Larvae	6	21	Corn	1200 ha, up to 0.4 larvae per plant and normally 9%–11% plants infested.
Hunan	April	Larvae	6	12	Corn	1300 ha, up to 1.2 larvae per plant and normally 5%–41% plants infested.
Hainan	April	Larvae	–	18	Corn	30 ha, up to 0.7 larvae per plant and 80% plants infested in Wanning county.
Fujian	May	Larvae	6	9	Corn	6.6 ha, up to 1.2 larvae per plant and 20%–40% plants infested.
Zhejiang	May	Larvae	2	2	Corn	5.3 ha, up to 0.02–0.1 larvae per plant and 1%–10% plants infested.
Hubei	May	Larvae	2	1	Corn	333 ha, up to 0.2–0.6 larvae per plant and 9.5%–18% plants infested in Tongshan county.
Sichuan	May	Larvae	1	1	Corn	<1 ha, up to 0.1 larvae per plant and 1.3% plants infested in Xichang City.
Jiangxi	May	Larvae	1	1	Corn	33 ha, up to 0.3 larvae per plant and 3%–30% plants infested in Ganzhou City.
Chongqing	May	Larvae	1	1	Corn	2 ha, up to 0.6 larvae per plant and up to 50% plants infested in Beibei District.
Henan	May	Larvae	1	1	Corn	<1 ha in Xinyang City.

#### Adult detections

In Yunnan from February 19 to April 1, 2019 in Ruili of Dehong, the total number of moths per sex pheromone traps and sticky‐card traps with food bait were 32 and 159, respectively. There were fewer moths, only 20, detected using vertical‐pointing searchlight traps from January to March from Jiangcheng of Pu'er. In Guangxi, the first *S. frugiperda* moth was detected in Hechi of Yizhou on March 11, 2019 (Table 2). Subsequently, more moths were trapped in Hechi (Du'an and Donglan counties), Baise (Youjiang and Tianlin counties) and Qinzhou (Pubei county) before larvae were detected in these regions.

### Molecular identification and phylogenetic analysis

**Figure 3:**
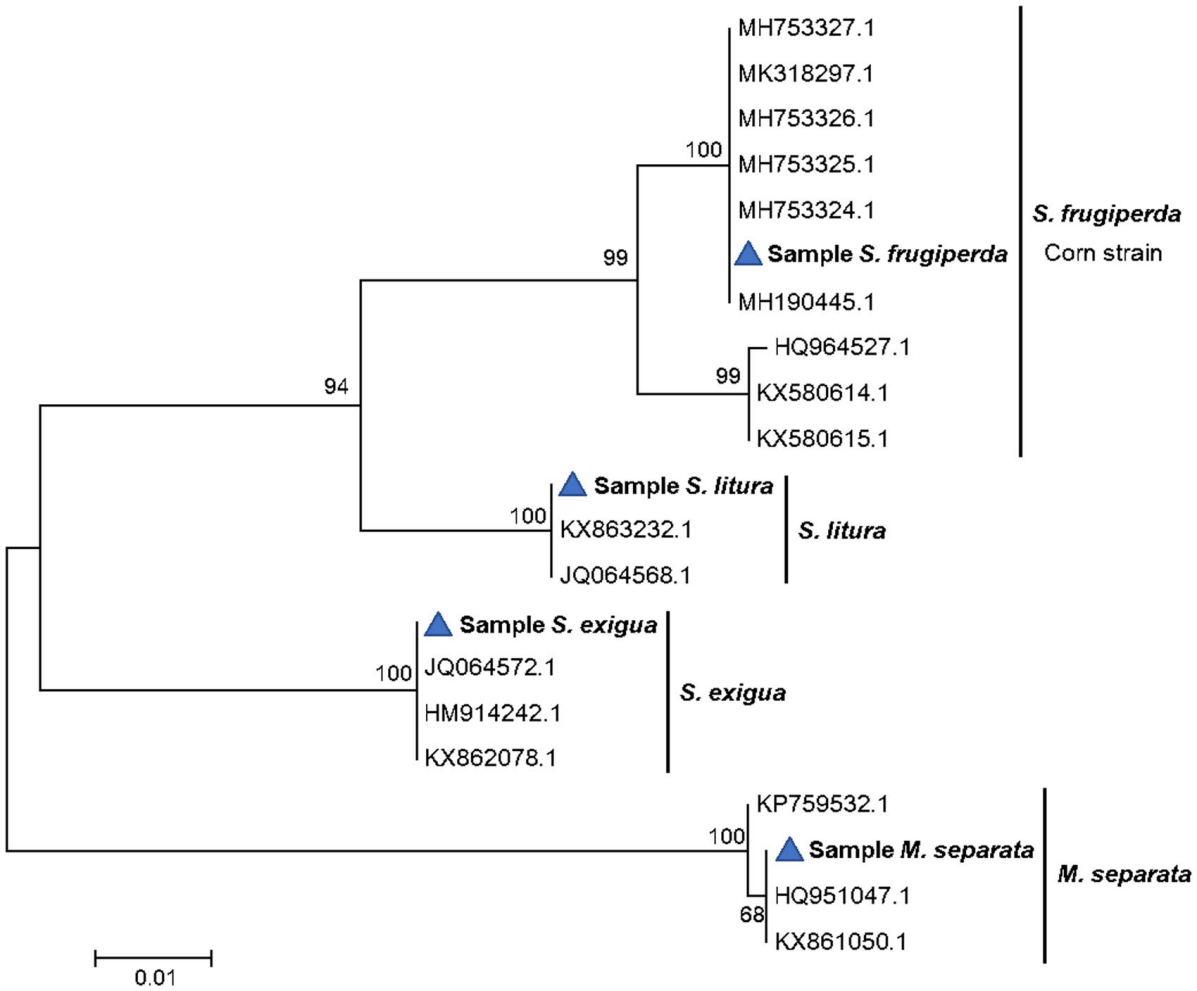
The phylogenetic tree was constructed using cytochrome oxidase subunit 1 (CO1) sequences from four Lepidoptera noctuid species. Maximum likelihood method was adopted based on the Tamura‐Nei model (Tamura & Nei, 1993). The nucleotide sequences were aligned using the Clustal W program and the evolutionary analyses were conducted in MEGA 7.0 (Kumar *et al*., 2016). The triangle indicates the samples collected from China used for this study.

The sequences obtained from our samples by CO1 marker were subjected to NCBI BLAST, the results showed positive hits and the species were identified as *S. frugiperda*, *S. litura*, *S. exigua* and *M. separata*. Especially, *S. frugiperda* from Yunnan of China belonged to a haplotype that showed 100% match with that of India (GenBank No. MH753324‐27 and MH190445). In addition, other lepidopterans showed sequence similarity above 90% when comparing with *S. frugiperda* (Fig. 3). Based on polymorphisms at *Tpi* e4_183_ site, 15 larval samples out of 20 belonged to the corn strain (*Tpi* e4_183_‐ C) and five samples showed heterozygous polymorphisms (*Tpi*‐h e4_183_ C and T).

## Discussion

The exotic *S. frugiperda* has been detected in some countries in Africa and Asia in recent years. The spread or invasion of *S. frugiperda* is not only related to global trade and transport, but also strong long‐distance migration ability of the *S. frugiperda* moths. Proactive actions for potential invasion of *S. frugiperda* to China coordinated by government agencies and agricultural extension systems resulted in rapid detection of the first larvae on January 11, 2019 in a cornfield in Yunnan province near Myanmar. Scientists, especially those from the Chinese Academy of Agricultural Sciences (CAAS), provided timely and important support on the invasive pest alert, migration forecasting, monitoring and identification methods, and pest management techniques (Guo *et al*., 2018; Guo *et al*., 2019; Wu *et al*., 2019; Zhang *et al*., 2019). The extensive monitoring in the southern provinces resulted in dynamic tracking of the *S. frugiperda* spreading in China, which is crucial for coordinating management of this pest in new locations. It was detected in >260 counties of 13 provinces within 4 months by May 10, 2019, suggesting it is spreading rapidly.

Wu *et al*. (2019) simulated the migration patterns from Myanmar to spread to Yunnan and Guangxi. Their flight toward other provinces in southern China (Guizhou, Guangdong, Hainan and Hunan Provinces) was also predicted. If prevention and control is not timely, the pest will be able to spread toward the main corn producing areas of China and even reach the Korean peninsula and Japan in June or July of 2019. Currently, *S. frugiperda* management mainly relies on the use of chemical insecticides and *Bacillus thuringiensis* (Bt) corn (Cook *et al*., 2004). However, with the repeated use of pesticides and highly adaptable capabilities of this species, *S. frugiperda* can develop resistance to those chemicals (Yu, 1991, Gutiérrez‐Moreno *et al*., 2019) and to the plants expressing a Cry1A or Cry1F Bt protein (Farias *et al*., 2014; Huang *et al*., 2014; Omoto *et al*., 2016). It is therefore essential to use an integrated pest management (IPM) approach to manage *S. frugiperda* and other Lepidoptera pests in corn. In March 2019, NATESC (2019d) published the IPM Guide on Fall Armyworm in China (2019 Preliminary Version), which will be updated based on current studies. Based on the experiences in North and South America (Huang *et al*., 2014; Burtet *et al*., 2017) and South Africa (Botha *et al*., 2019), Bt corn plants can be highly effective on *S. frugiperda* when utilized as part of an IPM program after registration for cultivation in China in the future. The use of pyramided traits expressing effective Bt proteins on *S. frugiperda* with different sites of action (Burtet *et al*., 2017; Marques *et al*., 2019) should be prioritized and then deployed with appropriate refuge, resistance monitoring, and grower education. In southern China, three similar noctuid larvae of native lepidopteran species (*S. litura*, *S. exigua* and *M. separata*) are often found in the same field and each species can cause considerable damage on important agricultural crops, such as corn, rice, wheat, cotton and vegetables (Jiang *et al*., 2011). However, on‐farm identification of larvae of these pests using morphological characteristics is challenging, and often results in misidentifications, especially for early instars. Moreover, the traditional morphological classification requires a high level of knowledge and experience by researchers or extension specialists. Therefore, a more accurate and timely identification method is needed to differentiate these species and that can be achieved through molecular characterization. In this research, four noctuid larvae of lepidopteran pests from cornfields with three of them belonging to the same genus were collected and identified by both morphology and molecular methods. From the morphological identification, we can separate *S. frugiperda* from the other three species (*S. litura*, *S. exigua *and *M. separata*). Molecular methods were used to further confirm the morphological identification results (Fig. 3). Fast and accurate molecular methods are very important for timely identifying and distinguishing the larvae with similar morphology in cornfields and designing the control measures on newly invaded species. Meanwhile, the molecular methods may help greatly to understand its distant migratory and invasion pattern around the globe.

Three native noctuid species (*S. litura*, *S. exigua *and *M. separata*) are important pests infesting corn in China. The invasive *S. frugiperda* has the potential to become a hugely important pest of corn, if not the most important pest of corn at least in certain regions of China. It is particularly problematic for corn plants because female moths oviposit on corn leaves and larvae have strong serrated mandibles that allow them to feed on most corn tissues (Brown & Dewhurst, 1975; Pogue, 2002). The cannibalistic nature of *S. frugiperda* larvae also is an important factor because it allows them to dominate interspecific competitors and reduce intraspecific rivals (Chapman *et al*., 2000). In addition, larvae usually bore into corn whorls or ears and develop under protected conditions, which allows them to avoid direct exposure to insecticides. This greatly increases the chance of survival for *S. frugiperda* during insecticidal sprays and low‐dose exposure to insecticides probably has contributed to their resistance to several classes of insecticides (Adamczyk *et al*., 1999; Gutiérrez‐Moreno *et al*., 2019).

Two *S. frugiperda* strains were identified, one preferentially infests corn and the other preferentially infests rice (Pashley *et al*., 1985). The rice strain also prefers to infest millet and grass and the corn strain prefers corn and sorghum (Nagoshi & Meagher, 2004a,b). Both strains can infest corn and are morphologically indistinguishable (Pashley, 1986). According to strain identification results, only corn strain moths were discovered in Yunnan province, China. The invasion path of *S. frugiperda* is likely India, Myanmar then Yunnan province, China, after which they migrated to the eastern and northern regions of China. This possibility is due to its long‐distance flight ability and adaptability to environments. Supporting our hypothesis, migration of *S. frugiperda* inside the American states and its adaptability to different climatic conditions are well documented (Raulston & Sparks, 1986). Sequencing results of CO1 are consistent with the sequence of *S. frugiperda* found in India, and this result strongly supports our hypothesis on the invasion path to China. China as the second largest corn growing region in the world has not yet approved commercial planting of Bt corns. As a new invasive species, it will take time to sort out the spread potential, migratory dynamics, and ultimately the impact this moth will have on crops in China. As a new invasion species, *S. frugiperda* will enhance its reproductive capacity and spreading performance as part of its life history strategy (Johnson, 1987). Hence, if *S. frugiperda* is not well controlled, it particularly may have a serious impact on corn production in China. In addition, there are other major pests of corn in China, including *Ostrinia furnacalis* (He *et al*., 2003), *Conogethes punctiferalis* (Lu *et al*., 2010) and *M. separata*. A sustainable and effective IPM strategy to mitigate the impact of these insect pests of corn, especially *S. frugiperda*, is urgently needed.

## Disclosure

The authors declare they have no conflict interests.
